# CKS1 expression in melanocytic nevi and melanoma

**DOI:** 10.18632/oncotarget.23648

**Published:** 2017-12-23

**Authors:** Anna A. Brożyna, Andrew Aplin, Cynthia Cohen, Grant Carlson, Andrew Joseph Page, Michael Murphy, Andrzej T. Slominski, J. Andrew Carlson

**Affiliations:** ^1^ Department of Tumor Pathology and Pathomorphology, Faculty of Health Sciences, Nicolaus Copernicus University Collegium Medicum in Bydgoszcz, Oncology Centre - Prof. Franciszek Łukaszczyk Memorial Hospital, Bydgoszcz 85-796, Poland; ^2^ Department of Cancer Biology, BLSB 524, Thomas Jefferson University, Philadelphia, PA 19107, USA; ^3^ Winship Cancer Institute, Emory University Hospital, Atlanta, GA 30322, USA; ^4^ Pancreas, Liver, and Cancer Surgery, Piedmont Healthcare, Atlanta, GA 30309, USA; ^5^ Department of Dermatology, UConn Health, Farmington, CT 06030, USA; ^6^ Department of Dermatology, Comprehensive Cancer Center, Cancer Chemoprevention Program, University of Alabama at Birmingham, Birmingham, AL 35294, USA; ^7^ Department of Pathology and Laboratory Medicine, Albany Medical College MC-81, Albany, NY 12208, USA

**Keywords:** melanoma, Skp2, ubiquitination, p27, prognosis

## Abstract

Cyclin-dependent kinase subunit 1 (Cks1) regulates the degradation of p27, an important G1-S inhibitor, which is up regulated by MAPK pathway activation. In this study, we sought to determine whether Cks1 expression is increased in melanocytic tumors and correlates with outcome and/or other clinicopathologic prognostic markers. Cks1 expression was assessed by immunohistochemistry in 298 melanocytic lesions. The frequency and intensity of cytoplasmic and nuclear expression was scored as a labeling index and correlated with clinico-pathological data. Nuclear Cks1 protein was found in 63% of melanocytic nevi, 89% primary and 90% metastatic melanomas with mean labeling index of 7 ± 16, 19 ± 20, and 30 ± 29, respectively. While cytoplasmic Cks1 was found in 41% of melanocytic nevi, 84% primary and 95% metastatic melanomas with mean labeling index of 18 ± 34, 35 ± 34, and 52 ± 34, accordingly. Histologic stepwise model of tumor progression, defined as progression from benign nevi to primary melanomas, to melanoma metastases, revealed a significant increase in nuclear and cytoplasmic Cks1 expression with tumor progression. Nuclear and cytoplasmic Cks1 expression correlated with the presence of ulceration, increased mitotic rate, Breslow depth, Clark level, tumor infiltrating lymphocytes and gender. However, other well-known prognostic factors (age, anatomic site, and regression) did not correlate with any type of Cks1 expression. Similarly, increasing nuclear expression of Cks1 significantly correlated with worse overall survival. Thus, Cks1 expression appears to play a role in the progression of melanoma, where high levels of expression are associated with poor outcome. Cytoplasmic expression of Cks1 might represent high turnover of protein via the ubiquination/proteosome pathway.

## INTRODUCTION

**Figure 1:**
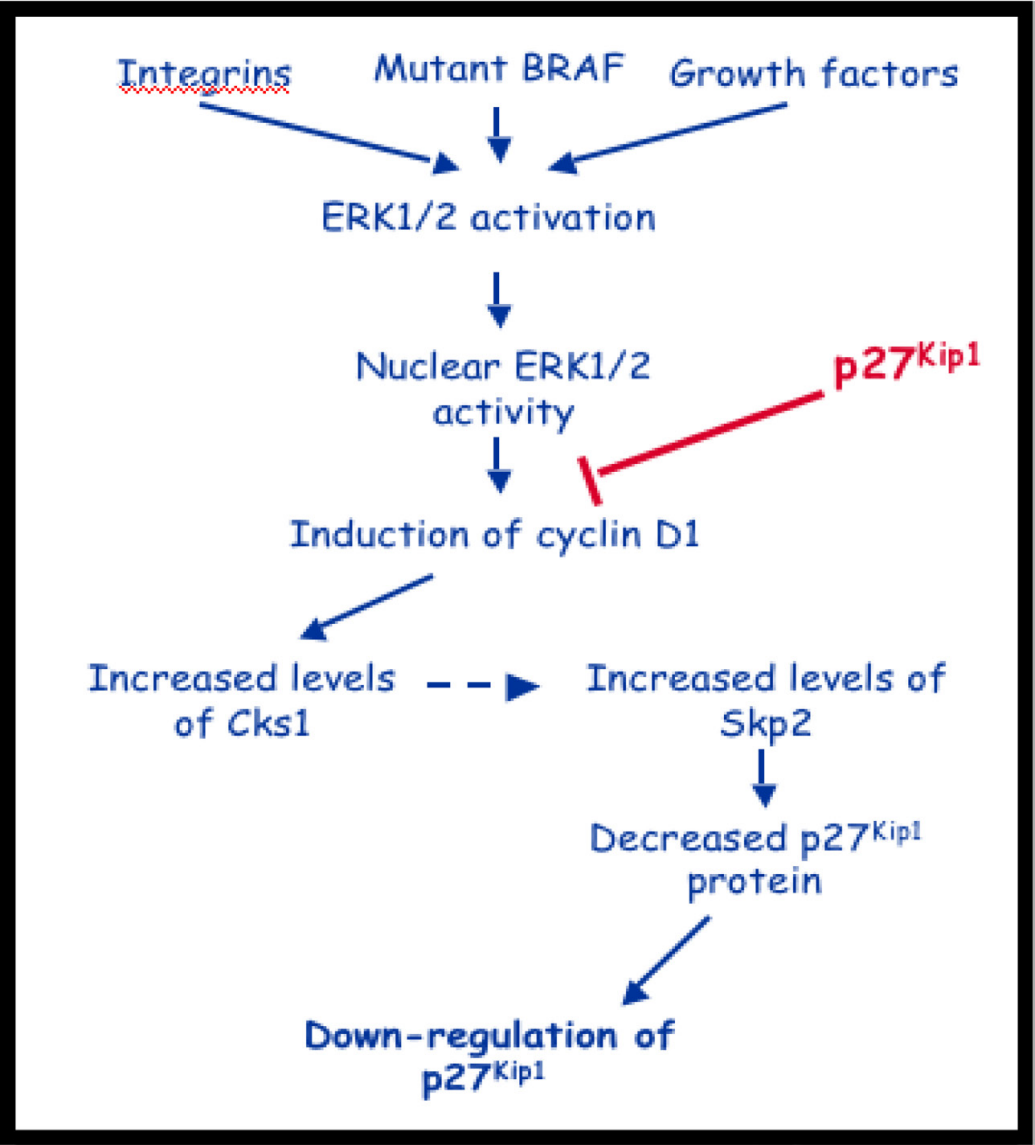
Putative role of Cks1 in pathogenesis of melanoma Constitutive activation of the MAPK pathway leads to increased Cks1 expression and down regulation of p27, a crucial G1-S cell-cycle regulator.

Cell cycle deregulation is a cornerstone of malignant transformation, leading to increased proliferation and growth rates. It is widely accepted that cell-cycle destabilization is common to many human malignancies. Induction of cellular proliferation is carried out in large part at the transcriptional level, where *cis-* and *trans-*activating factors induce the transcription of cell cycle progression mediators. Furthermore, ubiquitin ligase-mediated degradation of cell cycle progression factors represents a further level of regulation. It is this tight regulation of cell-cycle progression, which maintains tissue homeostasis. In normal tissue, cyclin-dependent kinase (CDK) activity is inhibited in the absence of mitogens, by the protein p27, via the ubiquitin proteasome pathway [1] (Figure 1).

The cyclin-dependent kinase subunit family is a highly phylogenetically conserved family of small (9-18kDa) proteins. There are two distinct orthologues, Cks1 and Cks2 in vertebrates [2, 3]. Cks1 is required in SCF^skp2^-driven p27 ubiquitinylation [2]. Cks1 induces an allosteric change in the Skp2 component of SCF^skp2^ ubiquitin ligase, allowing it to bind phosphorylated p27. This in turn allows for efficient transfer of the ubiquitin moiety, which, once bound to p27, leads to subsequent recognition by the proteasome [1, 4]. Cells lacking p27 degradation can progress abnormally through the cell cycle, because of unopposed CDK activity.

A likely mechanism for overexpression of Cks1, observed in many human malignancies [5–13], is *cis*-activation of the Cks1 gene promoter by proto-oncogenes such as Myc. Myc normally accelerates cell-cycle progression and its overexpression has been implicated in many tumors such as Burkitt’s lymphoma. Interestingly, quantitative mRNA expression analysis in Burkitt’s lymphoma-derived B-cells reveals increased Cks1 transcript levels, which suggests myc-dependent Cks1 overexpression and in turn increased p27 turnover, thereby allowing the malignant B-cells to transit through the cell cycle [14]. Cks1 also activates the transcription of factors responsible for cell-cycle progression and is associated with increased proliferation in several cell types [15].

Melanoma arises from malignant transformation of melanocytes and is one of the deadliest forms of skin cancer [5]. Melanocytes rely on adhesion factors to activate extracellular signal-regulated kinase 1/2 (ERK1/2), which, in turn induces cyclin D1 and down regulates p27 [16]. B-RAF regulates p27 mRNA abundance, independently of cyclin D1, and mutations of B-RAF are implicated in 70% of melanomas [17]. B-RAF controls p27 expression in melanoma cells at the mRNA level and at the level of proteasome degradation. The latter is cyclin D1-dependent and occurs via regulation of Cks1 and Skp2 [6]. B-RAF knockout mice showed decreased levels of cyclin D1 and increased levels of p27. Mice lacking Cks1 had lower Skp2 levels and an increase in p27 levels. Mice lacking both Cks1 and Skp2 harbored a further increase in p27 levels compared to Skp2 knockouts alone. Individual knockouts of either Cks1 or Skp2 significantly reduced cell growth [5]. This appears to support the hypothesis that B-RAF and cyclin D1 control expression of Cks1 and Skp2, which, in turn, mediate degradation of p27 in melanoma cells.

The most common mutation of B-RAF, V600E, hyper-activates B-RAF and the downstream mitogen-activated protein kinase (MEK)-ERK1/2 pathway. This pathway is an important regulator of G1-S progression. Indeed, several studies have shown that B-RAF^V600E^-MEK signaling is necessary for melanoma cell S phase entry, proliferation and anchorage-independent growth *in vitro* [18–22] and for melanoma cell sub-cutaneous growth in nude mice [21, 23]. Underscoring the importance of RAF-MEK-ERK1/2 signaling, this pathway may also be hyper-activated in melanomas expressing wild-type B-RAF through mutation of RAS, aberrant expression of G-protein coupled receptors and/or up-regulation of autocrine growth factors [24, 25]. It is critical to identify cell cycle markers that correlate with increased B-RAF signaling in melanoma since they may serve as tumor biomarkers, and alterations in their expression/regulation may underlie de novo or secondary resistance to B-RAF and MEK inhibitors.

Skp1-cullin1-F-box (SCF) E3 ubiquitin ligase complexes have been identified as important regulators of cell cycle progression [26, 27] as aberrant expression of Skp2 is an adverse prognostic sign in melanoma [28]. The F-box protein determines the substrate specificity of the complex. Skp2 is the F-box protein involved in SCF-mediated degradation of the cyclin-dependent kinase inhibitor, p27, during late G1 and S phase [4, 29–31]. Skp2 also targets p57, p21, cyclin E1 and origin recognition complex 1 (Orc1) for degradation [4, 30, 32–34]. Skp2 is required for melanoma cell growth, however in a manner independent of p27 but dependent on p53 [35]. In addition to SCF components, Skp2 requires a cofactor, cdc kinase subunit 1 (Cks1) [2, 4]. Recently demonstrated, mutant B-RAF-MEK signaling regulates Cks1 expression in melanoma cells [5]. Furthermore, Cks1 was found to regulate Skp2 expression and that Cks1/Skp2 complexes mediate B-RAF effects on p27^Kip1^ [5]. Importantly, earlier work on correlations between the pathological and immunohistochemical profiles in melanomas found that Skp2 cytoplasmic levels correlated with aggressive melanomas and predicted poorer 10-year survival rate [28].

Given the previously suspected role of Cks1 in regulating Skp2 [22, 28], herein, we investigated Cks1 Expression in a number of different melanocytic lesions, from common melanocytic nevi to metastatic melanoma to assess its role in melanoma progression and its impact on prognosis in primary melanoma patients.

## RESULTS

### Normal skin Cks1 expression

**Figure 2:**
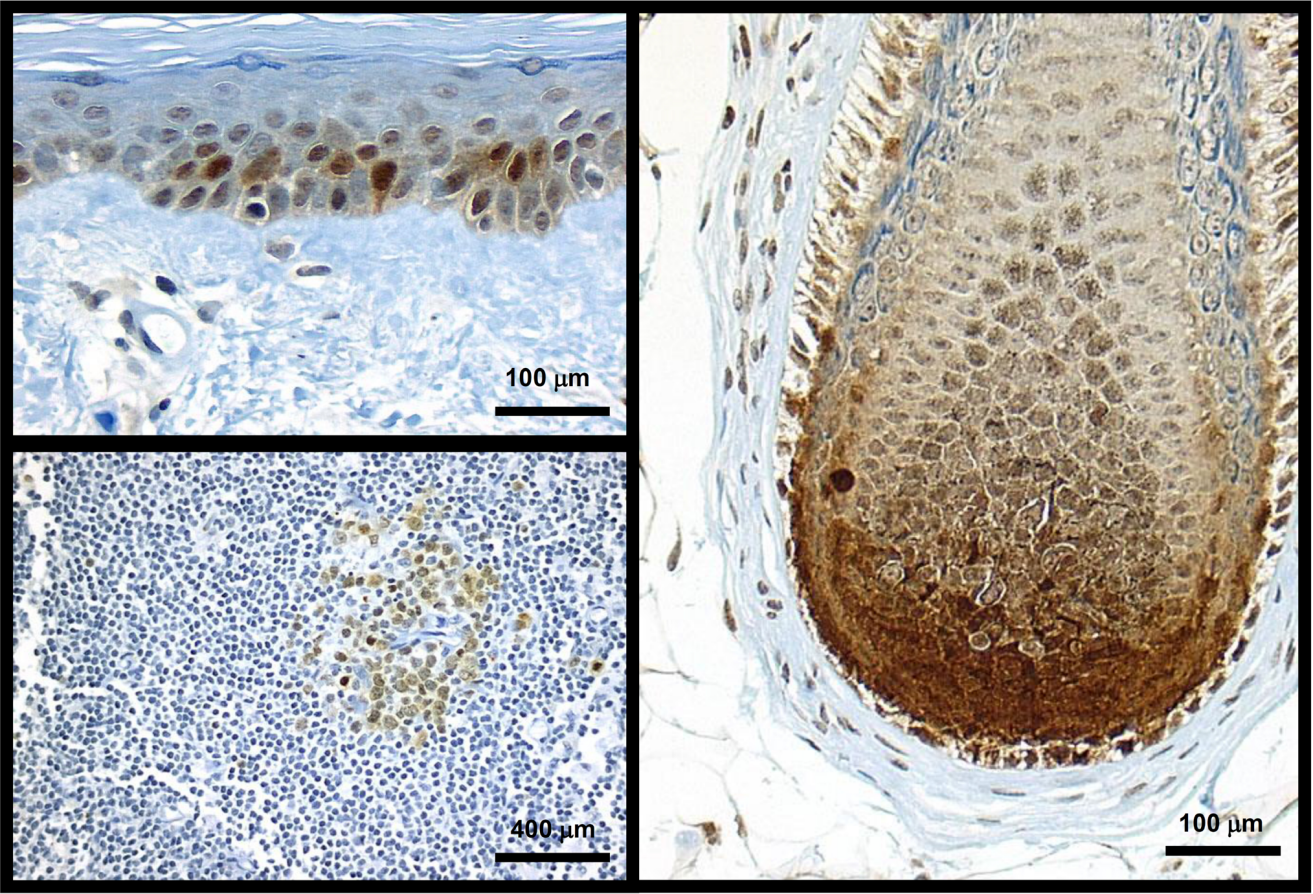
Cks1 expression in normal skin and lymph node was seen only in proliferating cells Sun-damaged skin with scattered keratinocytes showing Cks1 nuclear and cytoplasmic expression; normal melanocytes did not express Cks1 (top left). Follicular anagen bulb’s matrical keratinocytes exhibited intense nuclear and cytoplasmic expression of Cks1. Intervening dendritic melanocytes show faint nuclear and cytoplasmic Cks1 expression (right panel). Follicle center cells (bottom left) from a lymph node show low level of cytoplasmic and nuclear expression.

In control lymph nodes, lymphocytes within germinal follicles strongly expressed nuclear Cks1. Cks1 expression was assessed in the skin adjacent to melanocytic lesions (Figure 2). Table 1 summarizes these observations. Normal (resting) and hyperplastic epidermis (psoriasiform hyperplasia of epidermis adjacent to ulcers, overlying scars, or in regions of lichenification, eccrine coils, and follicular bulbs all showed strong, widespread nuclear Cks1 expression. However, only hyperplastic, not ‘resting’ keratinocytes exhibited cytoplasmic Cks1 expression. Sebocytes, follicular keratinocytes and smooth muscle cells of pilar erectus and media of vessels also expressed cytoplasmic Cks1. No Cks1 expression was seen in normal, junctional melanocytes.

**Table 1:** Cks1 expression in normal skin and lymphoid follicles

Normal skin constituents	Nuclear Cks1	Cytoplasmic Cks1
Adipocytes	+	+
Dermal scar/fibrosis	++	+/–
Eccrine ducts and coils	+	–
Epidermis, normal	+	–
Solar elastosis present	++	+/–
Hyperplastic epidermis	++	+
Follicular bulbar keratinocytes	+	+/–
Lymphoid follicles	++	++
Melanocytes	–	–
Nerves	–	+
Sebocytes	–	+
Smooth muscle cells	–	+
Vessels, endothelium	+/–	–

–:no expression; +/–:weak and variable expression; +:expression: ++: strong expression.

### Melanocyte phenotype and Cks1 expression

**Figure 3:**
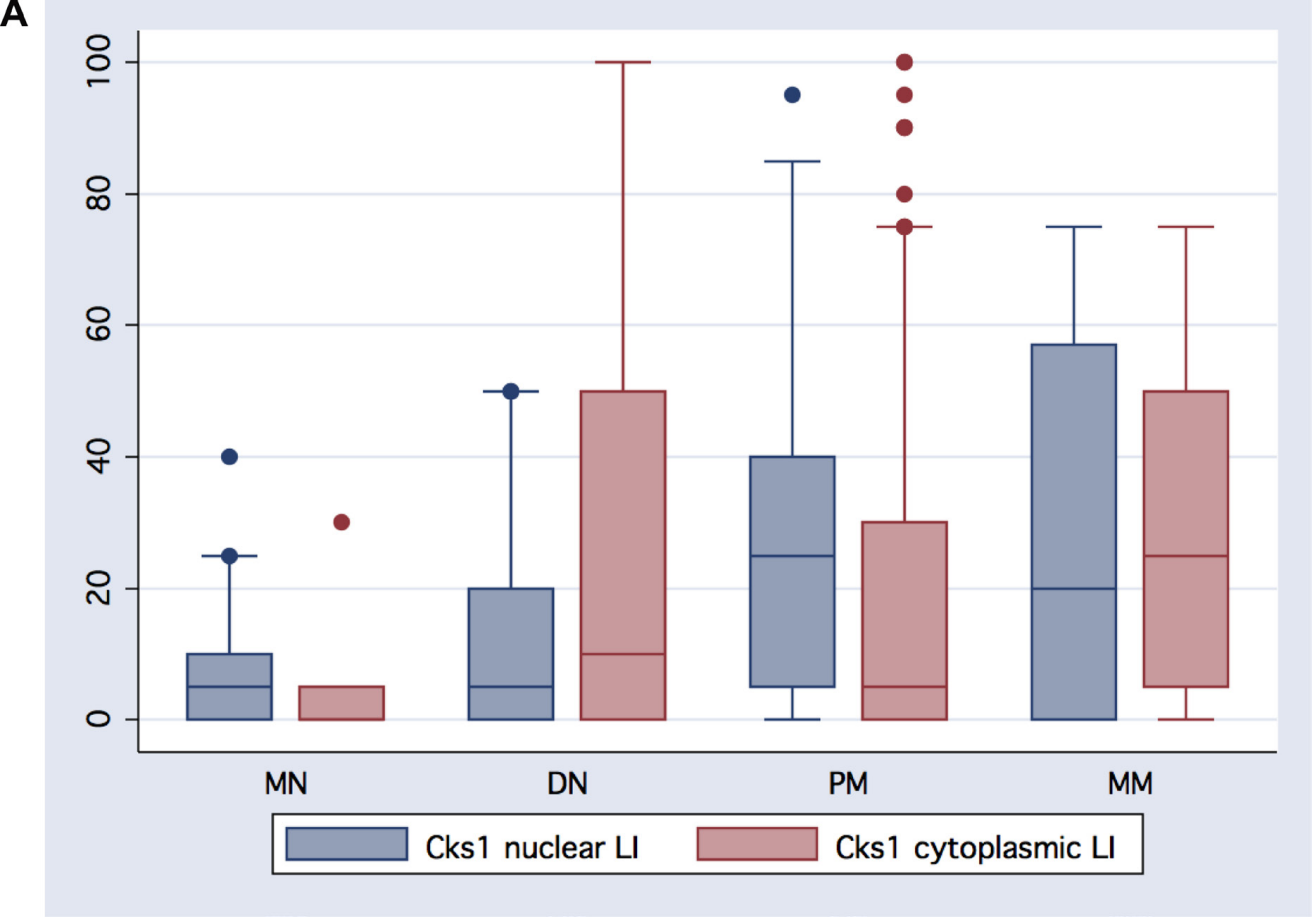

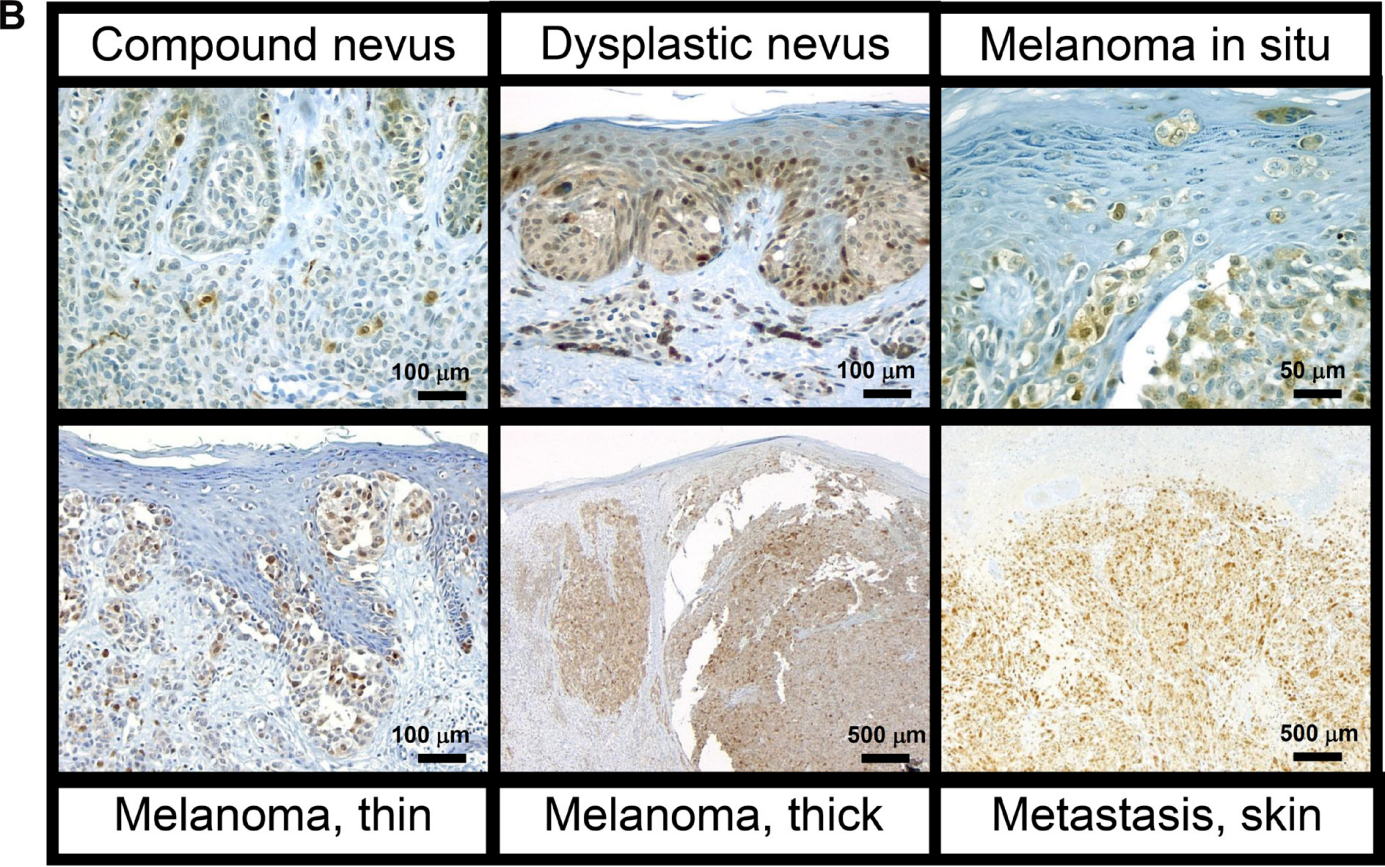
(**A**) Melanocytic lesions showed varying degrees of both cytoplasmic and nuclear Cks1 expression in the histologic stepwise progression: from banal common melanocytic nevi (MN) to dysplastic melanocytic nevi to primary melanoma (PM), and metastatic metastatic melanoma. (**B**) Increasing nuclear and cytologic Cks1 expression with melanoma progression.

Cks1 was found in melanocytic lesions and its expression was diverse by melanocytic nevus or melanoma cell cytologic phenotypes, or by pattern of growth. Uncommon nevi and dysplastic nevi showed some nuclear staining. Most melanocytic lesions, however, showed cytoplasmic staining. All melanoma showed variable nuclear staining (Figure 3).

### Quantification of Cks1 expression in melanocytic nevi, melanoma, and metastases

**Figure 4:**
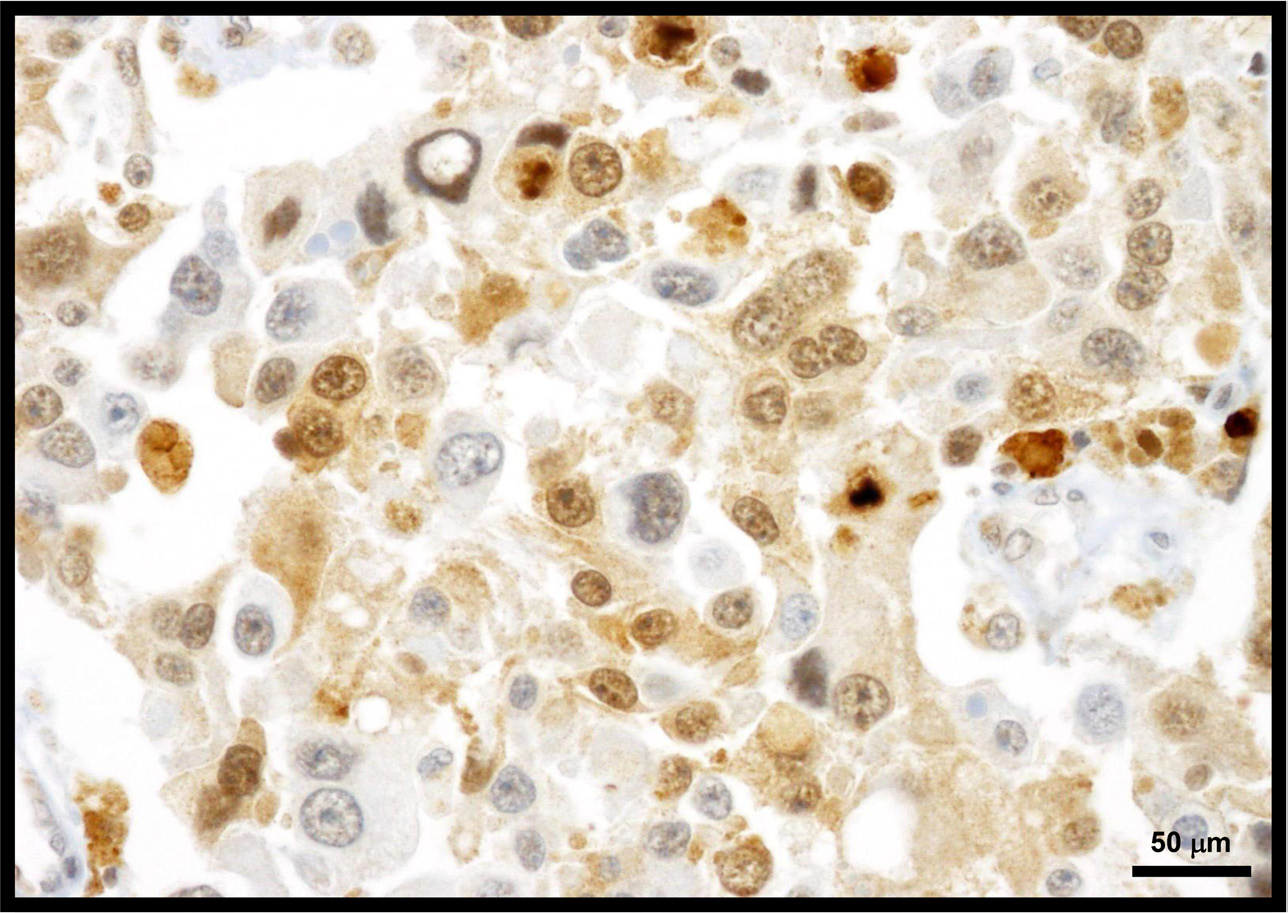
Melanomas typically express cytoplasmic and nuclear Cks1 protein Exhibited herein is a metastatic melanoma to lymph node showing predominate cytoplasmic Cks1.

**Figure 5:**
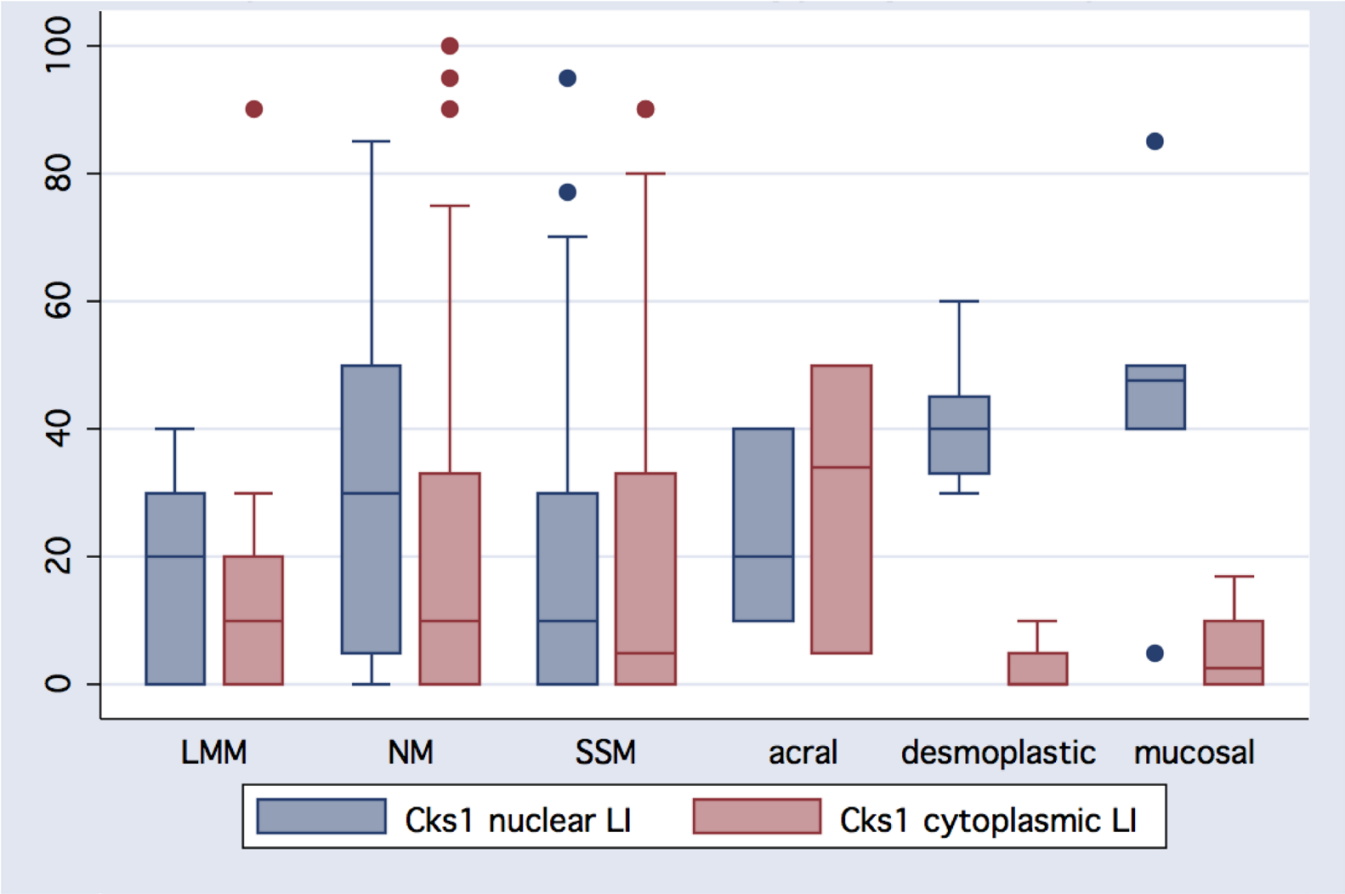
Heterogeneity of Cks1 nuclear and cytoplasmic expression amongst histologic melanoma subtypes Except for acral melanomas, nuclear expression exceeds cytoplasmic reactivity in different histological types of melanoma: LMM (lentigo maligna melanoma); NM (nodular melanoma); SSM (superficial spreading melanoma).

Table 2 summarizes the results for Cks1 overall expression and labeling indices for all melanocytic lesions by histologic classification (Figure 3, 4). Analysis of 123 primary melanomas with both *in situ* and invasive components revealed similar Cks1 expression in both components in 65 cases, higher Cks1 expression of *in situ* component in 38 cases and higher Cks1 expression in invasive component in 20 cases. Comparison of different histological subtypes of melanomas revealed highest Cks1 labeling index, especially nuclear staining, in nodular, desmoplastic and mucosal melanomas, indicating its correlation with aggressive histopathologic subtypes (Figure 5). Notably, expression of cytoplasmic Cks1 was significantly higher in primary melanomas from patients with a positive sentinel lymph node metastasis compared to primary melanomas from patients with a negative sentinel lymph node biopsy (55.7 ± 33.4 versus 36.6 ± 34.6, respectively, *p =* 0.01 for cytoplasmic Cks1 labeling index). However, no differences were noted for nuclear Cks1 labeling index in primary melanomas with or without an associated sentinel lymph node metastasis (19.6 ± 18.7 versus 23.8 ± 21.2, respectively, *p =* 0.21). Matched analysis of Cks1 expression in primary and metastatic melanomas from the same patients (*n =* 22) showed higher nuclear Cks1 labeling index in metastatic lesions in 13 cases, lower in 6 cases and similar expression in 4 cases (*n =* 23; in analyzed cohort one patient had two metastatic lesions evaluated by immunohistochemistry). Higher cytoplasmic Cks1 expression in metastatic melanomas was found in 13, lower in 9 and comparableCks1 expression in 1 patient.

**Table 2:** Cks1 expression in melanocytic nevi, primary cutaneous melanoma and metastatic melanoma

	Absolute cytoplasmic Cks1 expression (%)	Cytoplasmic Cks1 LI	Absolute nuclear Cks1 expression (%)	Nuclear Cks1 LI
Melanocytic nevi (*n =* 87)	17.7	18,1 ± 34,3 (0–100)	6.5	7.8 ± 15.9 (0–90)
Common acquired nevi (*n =* 33)	6.3	4.1 ± 8.5 (0–35)	3.6	8.2 ± 17.4 (0–90)
Dysplastic nevi (*n =* 29)	1.7	1.7 ± 4.1 (0–20)	1.0	3.1 ± 2.5 (0–5)
Spitz Nevi (*n =* 11)	45.0	45.0 ± 45.9 (0–100)	20.1	20.5 ± 26.1 (0–90)
Blue Nevi (*n =* 14)	63.8	63.9 ± 44.2 (0–100)	6. 0	6.4 ± 14.3 (0–50)
Melanoma *in situ* (*n =* 8)	10.4	11.3 ± 16.6 (0–50)	12.9	13.8 ± 11.9 (3–35)
Radial growth phase melanoma (*n =* 20)	22.3	22.3 ± 32.4 (0–100)	7.7	8.8 ± 22.0 (0–100)
Vertical growth phase melanoma (*n =* 143)	37.7	38.2 ± 33.4 (0–100)	21.5	21.9 ± 19.5 (0–90)
Nodular (*n =* 61)	46.1	46.3 ± 34.9 (0–100)	20.5	20.9 ± 18.3 (0–90)
Superficial spreading (*n =* 38)	28.6	29.1 ± 30.4 (0–100)	16.6	17.1 ± 18.9 (0–80)
Lentigo maligna (*n =* 20)	29.1	29,8 ± 32,8 (0–100)	26.4	27.0 ± 20.2 (0–70)
Acral (*n =* 10)	52.8	53,0 ± 35,4 (5–100)	19.2	19.5 ± 17.2 (5–60)
Mucosal (*n =* 6)	38.0	38.3 ± 18,6 (10–65)	39.2	39.2 ± 15.3 (10–50)
Metastatic melanomas (*n =* 40)	52.5	52.6 ± 33.8 (0–100)	30.2	30.5 ± 29.4 (0–100)
Lymph node (*n =* 26)	54.8	55.0 ± 34.5 (0–100)	32.0	32.3 ± 32.2 (0–100)
Skin/soft tissue (*n =* 6)	38.3	38.3 ± 24.2 (20–85)	31.8	32.5 ± 24.2 (10–75)
Visceral (*n =* 8)	55.4	55.6 ± 26.8 (20–100)	23.1	23.1 ± 24.6 (0–70)

LI: labeling index +cells/100 tumor cells reported as mean ± standard deviation; (range).

“Vertical growth phase melanoma” includes also desmoplastic (*n* = 6) and unclassified (*n* = 2) melanomas.

### Cks1 expression and melanoma progression

By linear regression methods, both nuclear and cytoplasmic Cks1 correlated with stepwise progression from melanocytic nevi to melanomas *in situ*, to primary melanomas, to metastatic melanomas (*r =* 0.34, *p <* 0.0001 and *r* = 0.32, *p <* 0.0001, respectively). With increasing American Joint Commission on Cancer (AJCC) tumor classification, nuclear and cytoplasmic Cks1 showed also significant positive correlation (*r =* 0.26, *p =* 0.0003, and *r =* 0.26, *p <* 0.0001, respectively). By AJCC stage (Ia, Ib, IIa, IIb, IIc, III (lymph node metastatic melanomas), and IV (visceral and distant skin metastatic melanomas)), nuclear and cytoplasmic Cks1 correlated with increasing AJCC stage (*r =* 0.22, *p =* 0.0024, and *r =* 0.36, *p <* 0.0001, respectively). See Table 3 for direct comparison of Cks1 expression with standard clinical and histologic prognostic markers.

**Table 3:** Comparison of Cks1 LI with standard prognostic markers

Category		Cytoplasmic Cks1 LI (mean ± SD)	Nuclear Cks1 LI (mean ± SD)
AJCC Tumor stage	T1a (*n =* 46)	21.52 ± 29.89	14.02 ± 23.87
	T1b (*n =* 3)	15.00 ± 18.03	6.67 ± 7.64
	T2a (*n =* 33)	33.64 ± 35.52	18.48 ± 18.14
	T2b (*n =* 13)	42.31 ± 33.83	20.38 ± 15.34
	T3a (*n =* 26)	36.35 ± 31.67	22.31 ± 14.51
	T3b (*n =* 15)	50.00 ± 28.22	32.00 ± 16.78
	T4a (*n =* 7)	47.14 ± 33.02	26.43 ± 32.24
	T4b (*n =* 20)	59.00 ± 31.44	26.25 ± 18.06
Clark Level*	Level 2 (*n =* 5)	23.00 ± 43.53	21.00 ± 44.22
	Level 3 (*n =* 31)	33.06 ± 33.71	11.77 ± 16.81
	Level 4 (*n =* 108)	34.81 ± 32.76	21.85 ± 18.64
	Level 5 (*n =* 18)	55.56 ± 31.24	26.67 ± 23.26
Ulcer	Absent (*n =* 116)	29.53 ± 32.43	17.67 ± 20.84
	Present (*n =* 47)	52.66 ± 30.82	26.81 ± 17.21
Mitotic counts/10hpf	0 (*n =* 62)	26.77 ± 35.00	12.90 ± 20.68
	1-4 (*n =* 52)	35.48 ± 32.39	20.48 ± 15.38
	>4 (*n =* 48)	49.90 ± 28.61	30.10 ± 20.51
Vascular invasion	Absent (*n =* 135)	35.19 ± 34.11	19.63 ± 20.31
	Present (*n =* 28)	41.07 ± 30.95	23.57 ± 19.90
Tumor vascularity*	Absent (*n =* 33)	29.24 ± 33.94	14.24 ± 23.56
	Sparse (*n =* 74)	36.76 ± 34.96	19.80 ± 18.57
	Moderate (*n =* 37)	44.32 ± 33.27	25.54 ± 21.76
	Prominent (*n =* 18)	31.94 ± 25.33	23.89 ± 14.30
Microsatellites	Absent (*n =* 153)	34.64 ± 32.64	19.87 ± 19.67
	Present (*n =* 10)	66.67 ± 36.31	30.0 ± 27.95
	Absent (*n =* 30-)	26.9 ± 25.8	44.4 ± 25.8
Tumor infiltrating lymphocytes			
	Non-brisk (*n =* 100)	22.0 ± 22.2	36.4 ± 33.4
	Brisk (41)	14.6 ± 27.9	27.9 ± 33.7
Regression	Absent (*n =* 126)	38.69 ± 34.24	20.28 ± 18.86
	Present (*n =* 36)	28.47 ± 30.11	20.97 ± 24.75
AJCC Stages I and II	Stage I (*n =* 82)	26.16 ± 32.28	15.55 ± 21.34
	Ia (*n =* 46)	21.52 ± 29.89	14.02 ± 23.87
	Ib (*n =* 36)	32.08 ± 34.63	17.50 ± 17.75
	Stage II (*n =* 81)	46.36 ± 31.92	25.12 ± 17.92
	IIa (*n =* 39)	38.22 ± 32.08	21.67 ± 14.61
	IIb (*n =* 22)	49.09 ± 29.06	30.23 ± 22.17
	IIc (*n =* 20)	59.00 ± 31.44	26.25 ± 18.06
Type of (1st) melanoma recurrence	Local (*n =* 6)	38.33 ± 34.22	32.50 ± 24.24
	Regional (*n =* 26)	55.00 ± 37.47	32.31 ± 32.16
	Distant (*n =* 8)	55.63 ± 26.78	23.13 ± 24.63
Outcome	Alive without disease (*n =* 76)	31.64 ± 34.35	15.07 ± 17.97
	Alive with disease (*n =* 15)	44.00 ± 33.60	29.33 ± 20.69
	Dead without disease (*n =* 21)	35.71 ± 35.96	20.71 ± 27.49
	Dead with disease (*n =* 45)	43.11 ± 29.97	27.00 ± 18.35

LI: labeling index +cells/100 tumor cells reported as mean ± standard deviation

*Per criteria of Kashani-Sabet M *et al*. [66]

NS – not statistically significant

### Comparison of Cks1 labeling index with histologic prognostic biomarkers

Both nuclear and cytoplasmic, Cks1 expression positively correlated with well-known prognostic factors (Tables 3 and 4). Specifically, the presence of ulceration (*r =* 0.21, *p =* 0.004 and *r =* 0.31, *p <* 0.0001, respectively), increasing mitotic rate (*r =* 0.33, *p <* 0.0001 and *r =* 0.27, *p =* 0.0002, respectively), tumor thickness (*r =* 0.2, *p =* 0.006, and *r =* 0.35, *p <* 0.0001, respectively), Clark level (*r =* 0.18, *p =* 0.011 and *r =* 0.17, *p =* 0.016, respectively). In addition, Cks1 expression negatively correlated with tumor infiltrating lymphocytes (for nuclear Cks1 labeling index *r =* –0.25, *p =* 0.0007, and for cytoplasmic Cks1 labeling index *r =* –0.18, *p =* 0.011). Cks1 nuclear labeling index correlated also with sex (*r =* 0.15, *p =* 0.028). However, other prognostic factors (age, anatomic site, vascular invasion and regression) did not correlate with nuclear or cytoplasmic Cks1 expression. There was no correlation between melanin pigmentation and Cks1 expression in radial growth phase. However, melanin content negatively correlated with nuclear Cks1 expression in vertical growth phase (Table 4). Furthermore, positive correlation between melanin pigmentation and both cytoplasmic and nuclear Cks1 expression in metastases was found (Table 4). Other relevant correlations of Cks1 expression with histopathological characteristics are presented in Table 4.

**Table 4:** Correlation of Cks1 with other clinicopathologic factors in melanoma patients

Category	Cytoplasmic Cks1 LI		Nuclear Cks1 LI	
	*r*	*p*	*r*	*p*
Age	NS	NS	NS	NS
Sex	NS	NS	0.15	0.028
Site	NS	NS	NS	NS
Breslow thickness	0.35	<0.0001	0.20	0.005
Clark Level	0.17	0.016	0.18	0.011
Ulcer	0.31	<0.0001	0.21	0.004
Mitotic counts/10hpf	0.27	<0.0001	0.33	0.0002
Tumor infiltrating lymphocytes	–0.18	0.011	–0.25	0.0007
Vascular invasion	NS	NS	NS	NS
Tumor vascularity	NS	NS	0.18	0.013
AJCC stage	0.36	<0.0001	0.26	0.0003
Microsatellites	0.22	0.0024	NS	NS
Regression	NS	NS	NS	NS
Associated nevus	–0.17	0.016	–0.16	0.018
Pigmentation-RGP	NS	NS	NS	NS
Pigmentation-VGP	–0.21	0.01	-0.33	0.0001
Pigmentation-metastases	0.36	0.03	0.34	0.04
Solar elastosis	NS	NS	0.17	0.018
Spindle melanocytes	NS	NS	0.17	0.023

RGP: radial growth phase; VGP: vertical growth phase

### Survival analysis and Cks1 expression

**Figure 6:**
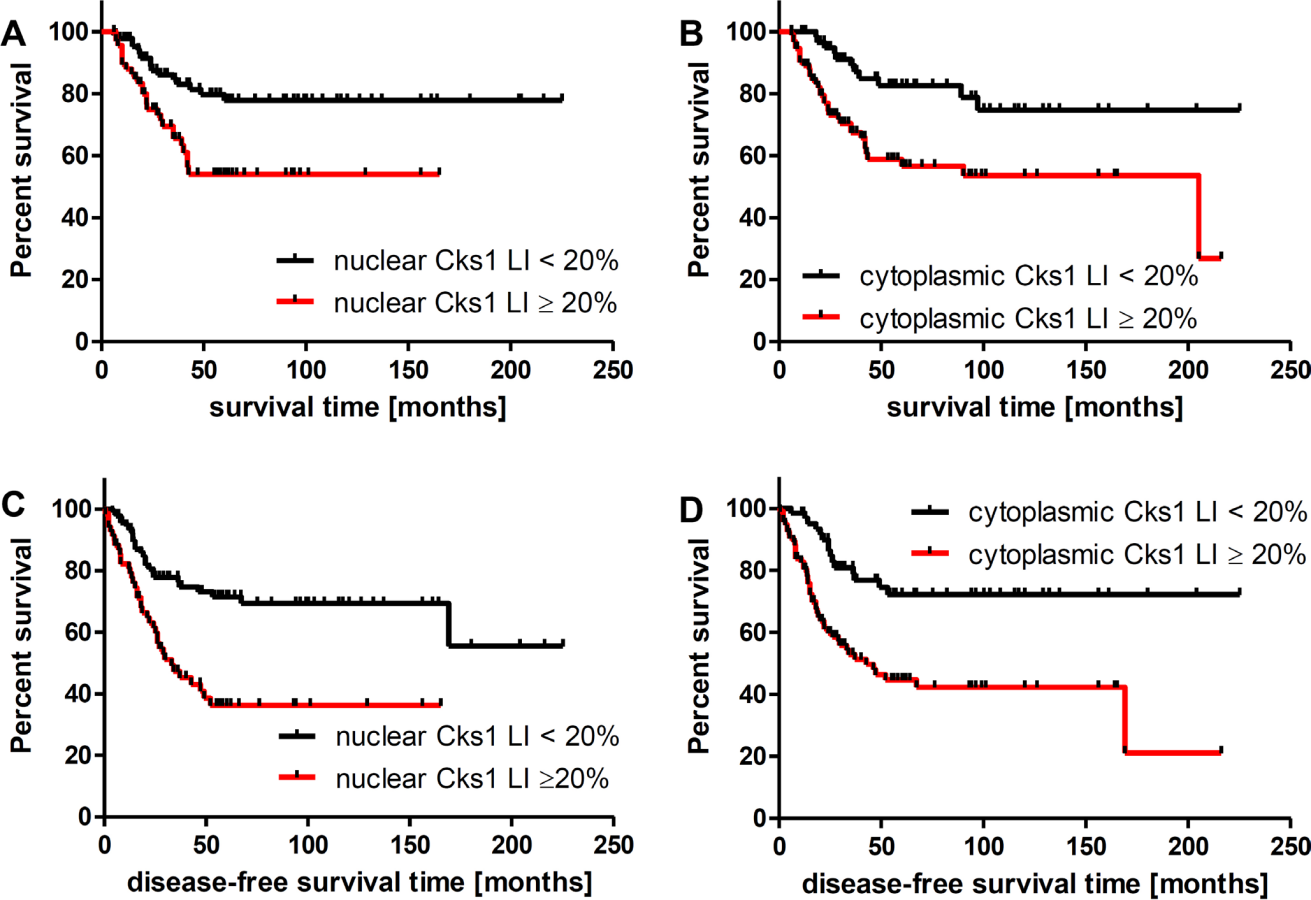
Five-year overall survival (**A**, **B**) and disease free survival (**C**, **D**). Significant decreased 5 year overall survival was associated with nuclear (A) and cytoplasmic (B) Cks1 labeling index of 20 or greater in primary melanoma (54% versus 78%, and 27% versus 75%, respectively). Significant decreased disease free survival was associated with nuclear (C) and cytoplasmic (D) Cks1 labeling index of 20 or greater in primary melanoma (36% versus 55%, and 21% versus 72%, respectively). LI - labeling index.

By univariate analysis, nuclear and cytoplasmic Cks1 labeling index (≥20) was found to be a predictor of poor disease-free and overall survival (Table 5, Figure 6). We also found that AJCC stage, depth, Clark’s level, ulceration, TILs, mitosis, vascularity and angioinvasion were predictors of disease-free survival and overall survival (Table 5). However, Cks1 expression was not an independent prognostic factor of disease-free survival and overall survival in melanoma patients on multivariate analysis. By multivariate analysis, age (Hazard ratio (HR) = 1.04; *p =* 0.001), vascular invasion (HR = 4.78; *p =* 0.0001) and AJCC stage (HR = 2.1; *p =* 0.003) were found to be independent overall survival factors.

**Table 5:** Predictors of prognosis by univariate analysis

	Disease-free survival			Overall survival		
	Hazard ratio	95% Conf. Interval	*p*	Hazard ratio	95% Conf. Interval	*p*
Age	1.01	1.00–1.02	*0.055*	1.05	1.02–1.07	**0.0001**
AJCC stage (I and II)	1.97	1.58–2.44	**0.0001**	1.93	1.49–2.50	**0.0001**
Angioinvasion	5.44	2.94–10.09	**0.0001**	6.23	3.10–12.50	**0.0001**
Associated nevus	0.79	0.42–1.47	0.46	0.97	0.48–1.99	0.64
Cks1 cytoplasmic (20%)	2.20	1.23–3.94	**0.008**	2.169	1.09–4.28	**0.027**
Cks1 nuclear (20%)	3.47	1.96–6.12	**0.0001**	3.51	1.80–6.85	**0.0001**
Clark’s level	4.33	2.36–7.97	**0.0001**	3.23	1.70–6.15	0.0001
Depth	1.34	1.19–1.51	**0.0001**	1.28	1.11–1.48	**0.001**
Mitosis	1.12	1.09–1.61	**0.0001**	1.11	1.06–1.15	**0.0001**
Pigmentation – stage I	2.698	0.6564–24.46	0.1326	2.567	0.4167–31.92	0.2424
Pigmentation – stage II	0.8327	0.4188–1.638	0.5882	0.7925	0.3481–1.735	0.5380
Pigmentation – stage III	1.166	0.4007–3.437	0.7705	0.9635	0.3537–2.620	0.9406
Pigmentation – stage IV	–	–	–	2.113	0.7163–10.60	0.1403
Regression	0.56	0.26–1.20	0.14	0.46	0.18–1.20	0.11
Sex	1.36	0.72–2.56	0.35	1.64	0.75–3.63	0.21
Site	1.00	0.77–1.28	0.97	0.64	0.70–1.27	0.68
TILS	0.98	0.97–0.99	**0.0001**	0.98	0.97–0.99	**0.003**
Tumor vascularity	1.82	1.38–2.40	**0.0001**	2.06	1.48–2.85	**0.0001**
Ulceration	3.97	2.25–7.02	**0.0001**	4.34	2.23–8.47	**0.0001**

TILS: tumor infiltrating lymphocytes. Bold significant (≤0.05; italics, trend (<0.10)

AJCC: American Joint Committee on Cancer. LI: labeling index.

## DISCUSSION

In this study, we found a significant increase in nuclear and cytoplasmic Cks1 expression with melanocytic tumor progression from melanocytic nevi to primary melanomas to metastatic melanomas. The results showed that overexpression of Cks1 in primary melanomas positively correlated with tumor advancement (Breslow thickness, Clark level, AJCC stage) and other well-known prognostic factors, such as gender, ulceration, mitotic counts, and negatively correlated with the presence of tumor infiltrating lymphocytes. In addition to nuclear expression of Cks1, we found also that cytoplasmic expression of Cks1, and its increasing cytoplasmic level, similarly to nuclear expression, correlated to melanocytic tumor progression, presence of poor-prognostic markers of melanoma, and worse survival.

Cks1 is ubiquitinated followed by proteasomal degradation, and some post-translational modifications, as observed in tumor cells, can modify Cks1 turnover [36]. Thus, increased cytoplasmic levels of Cks1 may represent high turnover of protein via the ubiquitination/protease pathway or its accumulation upon proteasomal blockade.

Cks1, as a cell control molecule, has been identified as a potential molecular target for cancer treatment since its silencing resulted in G2/M arrest and apoptosis of lung cancer cells without affecting normal fibroblasts [37] and growth inhibition of cells of oral squamous carcinoma [38]. Also reported, over expression of Cks1 correlated to radiotherapy resistance, both in patients, and in experimental cell-based study of esophageal cancer. In addition, over expression of Cks1 closely associated with poor pathological features of esophageal carcinoma, including higher histologic tumor grade, regional lymph nodes invasion, and neoplastic embolus [12, 13].

Increased cell proliferation rate is a common and important feature of malignant lesions, and in melanoma cells the deregulation of different mechanisms of cell-cycle control has been observed (reviewed in [39]. It was demonstrated that Cks1 is required for efficient Skp2-dependent p27 ubiquitination and its degradation [2, 4]. In different tumors, including melanomas, an inverse correlation between overexpression of Cks1 and reduced expression of cell-cycle inhibitor p27 has been shown [9, 14, 28, 40, 41]. Furthermore, decreased expression of p27 with the progression of tumors from nevi to melanomas, with lowest expression in metastatic melanomas, was reported [28, 42–44]. Some of these studies also revealed a negative correlation between reduced p27 expression and tumors thickness in nodular melanomas [42], as well as inverse correlation with disease-free survival, supporting the prognostic significance of cell-controlling p27 expression in melanomas. Other authors have not found such an association [28, 45]. In melanomas, similar to Cks1, increasing expression of Skp2 with melanocytic tumor development from nevi to primary and metastatic melanomas was also demonstrated [28]. Silencing of Skp2 resulted in inhibition of melanoma tumor growth *in vitro* and *in vivo* [46]. Elevated Skp2 expression similarly predicted worse 10-year overall survival [28].

The reduced survival related to overexpression of Cks1 has also been observed in other tumors, including multiple myeloma [41], nasopharyngeal [47], gastric [8], colorectal [9, 10], esophageal [12] cancers and others. Correspondingly, in some studies increased expression of Cks1 was related to other clinico-pathomorphological features, such as poor differentiation [11] and lymph node metastasis [13, 47]. In nasopharyngeal tumors, increased expression of Cks1 has also been identified as an independent poor prognostic factor [47]. Our findings are concordant with results of other studies, as we have shown that Cks1 expression is not only a predictor of disease-free survival, but also a predictor of overall survival. Higher expression of Cks1 is associated with reduced disease-free survival and overall survival in melanoma patients. Similarly, in our cohort, melanoma patients that developed metastases had elevated cytoplasmic Cks1 expression. Taken together, our findings point out the important role of Cks1 in melanoma biology and indicate the prognostic value of Cks1 in melanoma. In addition, Cks1 may be a potential molecule for targeted anti-melanoma treatment. However this concept should be confirmed using appropriate animal models including patient-derived orthotopic xenografts, which mimic clinical tumor growth and metastasis and have important clinical potential for melanoma patients [48–53]. Thus we also plan to perform an in depth mechanism oriented studies to define in details the role of CKS1 in melanoma progression and metastases and its usefulness as a target in anti-melanoma treatment using patient-derived orthotopic xenografts and transgenic mice.

Furthermore, this study showed relationships between melanoma stage and degree of tumor melaninization. First, a lack of relation between melanin pigmentation and Csk1 expression in radial growth phase melanomas; second, a negative correlation between nuclear Cks1 expression and vertical growth phase melanoma; and third, a positive correlation of melanin with nuclear and cytoplasmic Cks1 in melanized melanoma metastases. This spectrum suggests that melanogenic apparatus may affect the function and processing of Cks1 depending on the stage of tumor progression. It must be noted that melanogenesis may affect metabolic status and behavior of melanoma cells in a complex manner [54–59]. In addition, our previous study showed that higher melanin levels were associated with poorer prognosis and outcome following radiotherapy in melanoma patients [60, 61]. Animal-based study has revealed that spontaneous apoptosis of amelanotic melanoma cells is reduced in comparison to melanotic melanomas, and that pigmented melanoma cells are more resistant to induced apoptosis [62]. This is consistent, with experimental findings that inhibition of melanogenesis can act as sensitizer to radiation and cyclophosphamide induced toxicity or immune destruction of melanoma cells [63, 64]. Therefore, the potential effect of active melanogenesis or its intermediates on Cks1 expression and function deserves further careful and an in-depth investigation.

## CONCLUSIONS

In summary, nuclear expression of Cks1 increased with the stage of melanoma progression and correlated with poor overall survival. Cytoplasmic expression of Cks1 may represent high turnover of protein via the ubiquitination/protease pathway and is likely a marker of melanoma cell proliferation. These results indicate a role for Cks1 in the pathogenesis of melanoma. Further exploration of this topic will include multivariate survival analysis to determine if Cks1 is an independent prognostic variable and to evaluate whether Cks1 expression segregates with the presence or absence of specific gene mutations involved in melanoma such as BRAF and NRAS.

## MATERIALS AND METHODS

### Samples

Two hundred and ninety-eight paraffin blocks of melanocytic lesions were retrieved for this study from the files of the Department of Pathology, Albany Medical Center Hospital; Samuel Stratton Veterans Administration Medical Center; Emory University, Department of Pathology; and UConn Health. These samples consisted of 87 melanocytic nevi (9 junctional, 14 compound, 10 intradermal, 11 Spitz nevi, 29 dysplastic per criteria of Clemente *et al.* [67], and 14 blue nevi were examined), 8 melanoma *in situ*, 163 primary melanomas, and 40 metastatic melanomas (26 regional lymph node, 6 skin and soft tissue, and 8 visceral metastases). Clinicopathologic data were retrieved from reports (histologic diagnosis and melanoma histologic staging features were reviewed on each). Survival data were retrieved from the Tumor Registries at all 4 institutions. This study was approved by the institutional review board.

The primary melanomas patients had a mean age of 60 years (range 15–93 years old) and comprised of 53 females and 112 males (ages were not available for 6 patients). Clinical follow-up was available on 158 primary melanomas patients with a mean of 58 months of observation (range 6–225 months). Twenty-two of the primary melanomas patients had metastases available for immunohistochemical analysis. In addition, primary melanomas were classified by the updated AJCC tumor-node-metastasis and staging criteria [65]. The primary melanomas consisted of 46 stage Ia (46 T1a), 36 stage Ib (3 T1b, 33 T2a), 39 stage IIa (13 T2b, 26 T3a), 22 stage IIb (15 T3b, 7 T4a), and 20 stage IIc (20 T4b) melanomas.

### Immunohistochemistry

Expression of Cks1 (Zymed, San Francisco, CA; 1:50) was evaluated by immunohistochemistry. The frequency and intensity of cytoplasmic and nuclear expression was scored as a labeling index. This data was correlated with clinical, pathologic, and outcome data. Melanocytic nevi served as a control. Non-tumor elements (sweat glands, lymphocytes) also served as a control and exhibited Cks1 staining.

### Statistical analysis

Statistical analysis was carried out with the STATA (College Station, TX) statistical package. Differences between groups were tested by the chi-squared test for dichotomous variables and by the Student’s *t*-test for continuous variables. Correlations between study variables were examined by linear regression and pairwise covariance methods. Survival analysis was performed using the Kaplan Meier method. In addition, univariate and multivariate Cox proportional hazards models were applied to assess the effect of CKS1 expression and other variables and disease-free survival and overall survival. GraphPad Prism 5.0 (La Jolla, CA) was used to prepare survival graphs. The criterion for significance was *p ≤* 0.05.

## Abbreviations

AJCC: American Joint Commission on Cancer
CDK: cyclin-dependent kinase
Cks1: cyclin-dependent kinase subunit 1
ERK1/2: extracellular signal-regulated kinase 1/2
MAPK: mitogen-activated protein kinase
MEK: mitogen-activated protein kinase kinase
Skp2: S phase kinase-associated protein 2
